# Development of a mobile application for urinary diary monitoring in overactive bladder management

**DOI:** 10.1590/1806-9282.20250174

**Published:** 2025-10-27

**Authors:** Rüveyda Ölmez Yalazı, Nurdan Demirci

**Affiliations:** 1 Marmara University, Institute of Health Sciences, Department of Obstetrics and Gynecology Nursing – Istanbul, Türkiye.; 2 Çanakkale Onsekiz Mart University, Faculty of Health Sciences, Division of Midwifery – Çanakkale, Türkiye.; 3 Marmara University, Faculty of Health Sciences, Department of Obstetrics and Gynecology Nursing, Division of Nursing – Istanbul, Türkiye.

**Keywords:** Overactive bladder, Mobile application, User-centered design

## Abstract

**OBJECTIVE:**

The aim of this study was to develop a mobile application for the management of overactive bladder by means of urinary diaries. The application was designed to enhance usability and user satisfaction, with the Analyze, Design, Develop, Implement, Evaluate model serving as the overarching systematic framework.

**METHODS:**

The Analyze, Design, Develop, Implement, Evaluate model guided the development process, starting with analyzing patient and professional needs. User-centered mockups were designed, leading to a functional prototype. The app was developed, tested through assigned tasks, and evaluated for usability using scales, qualitative data, and feedback.

**RESULTS:**

Most healthcare professionals and patients found the tasks "very easy" or "easy." Average completion times were 13 min for patients and 9 min for professionals. Participants gave positive feedback, describing the application as intuitive and easy to use. No critical errors were identified, and satisfaction levels were high.

**CONCLUSION:**

The mobile app showed high usability and user satisfaction, demonstrating its potential to enhance overactive bladder management and improve patient care efficiency.

## INTRODUCTION

Overactive bladder (OAB) is characterized by urinary urgency, often accompanied by frequency or nocturia, without urinary tract infection or other identifiable diseases^1^. Its prevalence ranges from 12 to 22% in Europe and 16 to 17% in the United States, with a 2014 study in Turkey reporting urgency urinary incontinence (UI) prevalence at 29.3%^2–4^. While prevalence is similar in men and women, certain OAB symptoms are more frequent in women due to anatomical and physiological differences^5,6^. OAB significantly impacts daily life, including work, social interactions, sleep, and sexual functions, with women experiencing greater sleep disturbances, reduced sexual satisfaction, and lower quality of life^5–7^.

Treatment for OAB includes pharmacological, non-pharmacological, and surgical options, following stepwise guidelines by the American Urological Association (AUA) and the Society of Urodynamics, Female Pelvic Medicine, and Urogenital Reconstruction (SUFU)^8–10^. Non-pharmacological methods, such as bladder training, are favored due to concerns over medication side effects and invasive procedure risks^11^. Mobile applications offer a cost-effective solution for improving treatment adherence and sustainability by providing education and follow-up^12,13^.

This study develops and evaluates Turkey's first mobile app for urinary diary monitoring in women with OAB. Based on the ADDIE (Analyze, Design, Develop, Implement, Evaluate) model, the app integrates bladder training, reminders, and educational content. Usability and user satisfaction will be assessed to improve clinical management and patient outcomes.

## METHODS

This study employed a user-centered design approach guided by the ADDIE model to develop and test a mobile application for urinary diary monitoring in patients with OAB. Participants were recruited using the snowball sampling method^14^. Initial participants were identified through professional networks, and additional participants were referred by these initial recruits.

The sample included two groups:

Healthcare professionals: This group comprised 10 gynecology physicians and six gynecology nurses (n=16), all with at least 6 months of experience in urogynecology.

Patients: The patient group comprised 10 women diagnosed with OAB, aged 18 years or older, literate, and willing to participate in usability testing.

Ethical approval was obtained from Canakkale Onsekiz Mart University's Ethics Committee (approval number: 2024-207). Participants were provided with an information sheet and consent form before accepting the invitation to participate. All consent forms were obtained before the commencement of the study.

The ADDIE model, comprising Analysis, Design, Development, Implementation, and Evaluation, provides a flexible framework for designing educational tools^14^. In the Analysis phase, two user profiles—healthcare professionals and patients—were defined, shaping the app's data architecture. The Design phase involved mockup creation, usability testing, and satisfaction assessment. During Development, mockups were refined, and usability testing environments were prepared. The Implementation phase included usability testing with 14 health professionals and 10 OAB patients. Recorded sessions captured user feedback. The Evaluation phase analyzed usability and satisfaction, refining the app further.

Inclusion criteria for healthcare professionals required at least 6 months of experience in gynecology, while patients needed to be literate, over 18 years old, diagnosed with OAB, and provide consent to participate. The mobile application usability scale (MAUS), adapted to Turkish culture, evaluated usability through 40 items across 10 dimensions on a 7-point Likert scale^15,16^. Participants used a smartphone prototype to complete 6–7 tasks. Success rates, task times, and usability issues were recorded via the "think-aloud" method and anonymized transcripts. Observations and hospital follow-ups ensured a user-centered design process.

The quantitative data obtained were analyzed using SPSS version 22. The researchers manually analyzed the qualitative data from the usability tests without using any software tools.

## RESULTS

The application's intuitiveness was assessed using the think-aloud method. The researchers transcribed audio recordings and extracted key statements to identify design improvements. Two out of 14 professionals had difficulties with account creation and two suggested a more prominent section. The same difficulties were reported by six out of 10 patients. Most professionals (12 out of 14) found the test simple, as did nine of 10 patients (Table 1). All participants noted that data entry could be done from any environment and that they could complete tasks without prior training. Table 2 presents participant feedback collected via the think-aloud method and difficulties identified in tasks.

**Table 1:** Participants’ successful completion rates for tasks.

Participants		Yes		No	
Healthcare professionals	Task	n	%	n	%
	Create an account	12	86	2	14
	Log in	14	100	0	0
	Enter patient information	14	100	0	0
	Select a specific date range	14	100	0	0
	View patient data entries	14	100	0	0
	Provide feedback to the patient	14	100	0	0
	If the patient has asked a question, respond to it	14	100	0	0
Women diagnosed with an overactive bladder	Create an account	7	70	4	40
	Log in	8	80	2	20
	Select and save urinary diary data	8	80	2	20
	If the patient forgot to add urinary diary data, save it through the later entry tab	8	80	5	50
	Enter the bladder training section at any desired time	10	100	0	0
	Ask a question in the "Ask a Question" section	10	100	0	0

**Table 2:** Participants’ comments collected through the think-aloud method and difficulties identified in tasks.

Participants	Participants’ comments	Task difficulties
Healthcare professionals (H)	"It was quite simple to view the patients’ data, but some patients may enter incorrect data, so I might have to verify the data." (H4)	They were concerned about encountering incorrect data entries, but they were able to correct them quickly.
	"Reviewing the daily reports was easy, but it was time-consuming as I had to open each patient's data separately in the app." (H11)	During the data review, checking each patient's data individually caused a time management challenge.
	"The amounts and frequencies of urine entered by the patients were easily visible, which sped up the evaluations." (H6)	They saw that they could monitor the patients’ conditions without difficulty in accessing the data.
	"Some patients leave gaps in their data entries, which can cause confusion in treatment planning." (H1)	They were concerned about missing information in the treatment process due to incomplete data entry but felt relieved upon seeing the "Ask a Question" section.
Women diagnosed with an overactive bladder (W)	"When I first opened the app, I was confused about how to fill out the urinary diary, but the guide menu directed me." (W5)	Initially, they felt a lack of guidance but resolved the issue with in-app navigation.
	"It was a bit difficult to find the time slot for urine entries, but after getting used to it, the process became easier." (W2)	At first, they had difficulty selecting the time slot, but they adapted quickly.
	"Using the app regularly every day was challenging, but the reminder notifications made it easier." (W8)	They felt a lack of motivation for daily use but overcame this challenge with reminders.
	"Switching between options during data entry can be difficult, but overall, I found it practical to use." (W3)	"They experienced some confusion with certain options during data entry, but overall, the user experience was positive."

In usability tests, over 80% of healthcare professionals rated most tasks as "very easy," with none rating any as "difficult or very difficult." In the patient group, over 80% also rated these tasks as "easy." No tasks were marked as "difficult or very difficult."

Six tasks were completed on the first attempt by the professional group: logging in, entering patient information, selecting a date range, viewing data entries, providing feedback, and answering questions. The account creation success rate was 86% (Table 1). Professionals suggested simplifying information presentation to optimize decision-making time, recommending summary and detailed lists on the monitoring page. Dates were entered reluctantly.

All participants in the patient group successfully completed bladder retraining and question-related tasks. The success rates for creating an account, logging in, entering urinary diary data, and making subsequent entries were 70, 80, 80, and 80%, respectively (Table 1). Neither group made critical errors. MAUS results showed participants rated the app as "usable," with an overall MAUS score of 6.08±1.25 for healthcare professionals and 6.01±1.32 for women with overactive bladder. The highest sub-dimension scores were observed in control, shape, and animation balance. Additional ratings were given for hierarchy (24.05), entry point (25.36), fingertip size control (25.16), shape (24.96), animation balance (24.54), and transition (24.05). The highest scores were obtained for color and control, while the lowest scores for font (Table 3). Average task completion times were 13 min for patients and 9 min for professionals.

**Table 3:** Mobile application usability scale.

Scale sub-dimensions and total value	Healthcare professionals	Women diagnosed with an overactive bladder
Esthetic graphics	6.45±1.35	6.36±1.24
Color	6.16±1.24	6.16±1.24
Control	6.36±1.24	6.36±1.24
Font	5.87±1.19	5.51±1.53
Hierarchy	6.16±1.24	6.05±1.35
Entry point	6.36±1.24	6.16±1.24
Fingertip size control	6.45±1.35	6.36±1.24
Integrity	6.16±1.24	6.36±1.24
Shape	6.45±1.35	6.36±1.24
Animation balance	6.45±1.35	6.36±1.24
MAUS	6.08±1.25	6.01±1.32

MAUS: the mobile application usability scale.

## DISCUSSION

This study provides information about Bladder Guide, an m-health app that connects women who maintain urinary diaries with healthcare professionals. It provides real-time feedback, helping with symptom tracking, customizing treatment plans, and improving compliance. It has been proven to be valid, reliable, and usable. The application's design is to streamline data.

Research shows that women with UI have a positive attitude toward m-Health, as it enhances care access and protects their privacy^17^. No studies in Turkey have investigated the use of urine diaries in this way. Mobile applications without scientific evidence can also undermine trust, reducing benefits in care^18^. For instance, participants found real-time data entry particularly useful, supporting the literature that suggests mobile apps reduce memory bias^19^. Therefore, it is crucial for mobile applications to be developed through a user-centered and scientifically rigorous process to gain the trust of healthcare professionals and ensure reliable outcomes.

The development of our application was guided by criteria for evaluating urinary diaries, incorporating feedback from nurses and doctors^20^. It confirms content and criterion validity, showing high correlations between paper and app measurements of OAB symptoms. Mobile apps can help patients manage chronic conditions such as UI, improving their quality of life^21^.

The mobile application was believed to have strong usability due to the user-centered approach employed during its development^21^. Specialist doctors, nurses, and women diagnosed with OAB were involved from the outset. This interactive process addressed user needs and expectations regarding functionality and design, continuing until users were satisfied. However, this study's sample size was relatively small (n=26), which limits the generalizability of the findings. Future studies with larger and more diverse populations are needed to validate the results and examine subgroup differences (e.g., between nurses and physicians). Usability was evaluated through a mixed-methods approach using performance indicators, interviews, and validated surveys. A limitation of this study is the lack of long-term follow-up, which means it is unclear how effective the app is at keeping users engaged and improving clinical outcomes. Longitudinal studies would be useful here. Some patients experienced difficulties creating an account and selecting a time slot, showing that the interface needs improvement. This should include guided tutorials and improved ways to input data. Although a manual content review was used due to the limited data, future evaluations will include structured thematic analyses using qualitative software to extract broader usability patterns.

Compared to other mobile app usability studies, this study shares similarities and differences for both healthcare professionals and women with an OAB. Similar to Wadensten et al.'s study on the Tät II app, both groups rated controllability highly^22^. In addition to the Tät II app, comparative evaluations with tools such as MyPFD and OAB Journal could help contextualize the unique features and performance of this application. However, women with an OAB gave lower scores for visual elements, similar to Wildenbos et al.'s findings with older users in m-Health apps^23^. Additionally, as Zhao et al. noted, differences between user experiences suggest the need for customization and improvement^24^. Recent studies exploring pelvic organ prolapse interventions and pessary comparisons further emphasize the need for individualized, evidence-based mobile health solutions in pelvic floor care^25,26^. Healthcare professionals’ higher scores for control and esthetics may reflect their familiarity with digital health tools, underscoring the need to create apps that cater to a wider range of users.

This study, with 24 participants, relied on qualitative and quantitative evaluations. While sufficient for the methodology, further research is needed to assess long-term real-world use. Strengths include early user involvement, professional feedback, and robust usability, reliability, and validity assessments.

## CONCLUSION

The application offers clinical and educational benefits, enabling accurate symptom monitoring, bladder training resources, and improved self-management. It supports targeted, evidence-based care and integrates patient education with clinical management. Future updates may include interactive modules, gamification, and training programs for healthcare professionals to enhance engagement and impact.

## Data Availability

The datasets generated and/or analyzed during the current study are available from the corresponding author upon reasonable request.
